# Myocyte Enhancer Factor 2A Plays a Central Role in the Regulatory Networks of Cellular Physiopathology

**DOI:** 10.14336/AD.2022.0825

**Published:** 2023-04-01

**Authors:** Benrong Liu, Wen-Chao Ou, Lei Fang, Chao-Wei Tian, Yujuan Xiong

**Affiliations:** ^1^Department of Cardiology, Guangzhou Institute of Cardiovascular Disease, Guangdong Key Laboratory of Vascular Diseases, State Key Laboratory of Respiratory Disease, the Second Affiliated Hospital, Guangzhou Medical University, Guangzhou, China.; ^2^General Practice, the Second Affiliated Hospital, Guangzhou Medical University, Guangzhou, China.; ^3^Department of Laboratory Medicine, Panyu Hospital of Chinese Medicine, Guangzhou University of Chinese Medicine, Guangzhou, China.

**Keywords:** myocyte enhancer factor 2, MEF2A, MEF2C, MEF2D, GLUT4, CaMK

## Abstract

Cell regulatory networks are the determinants of cellular homeostasis. Any alteration to these networks results in the disturbance of cellular homeostasis and induces cells towards different fates. Myocyte enhancer factor 2A (MEF2A) is one of four members of the MEF2 family of transcription factors (MEF2A-D). MEF2A is highly expressed in all tissues and is involved in many cell regulatory networks including growth, differentiation, survival and death. It is also necessary for heart development, myogenesis, neuronal development and differentiation. In addition, many other important functions of MEF2A have been reported. Recent studies have shown that MEF2A can regulate different, and sometimes even mutually exclusive cellular events. How MEF2A regulates opposing cellular life processes is an interesting topic and worthy of further exploration. Here, we reviewed almost all MEF2A research papers published in English and summarized them into three main sections: 1) the association of genetic variants in MEF2A with cardiovascular disease, 2) the physiopathological functions of MEF2A, and 3) the regulation of MEF2A activity and its regulatory targets. In summary, multiple regulatory patterns for MEF2A activity and a variety of co-factors cause its transcriptional activity to switch to different target genes, thereby regulating opposing cell life processes. The association of MEF2A with numerous signaling molecules establishes a central role for MEF2A in the regulatory network of cellular physiopathology.

## 1. Introduction

**Figure 1.**
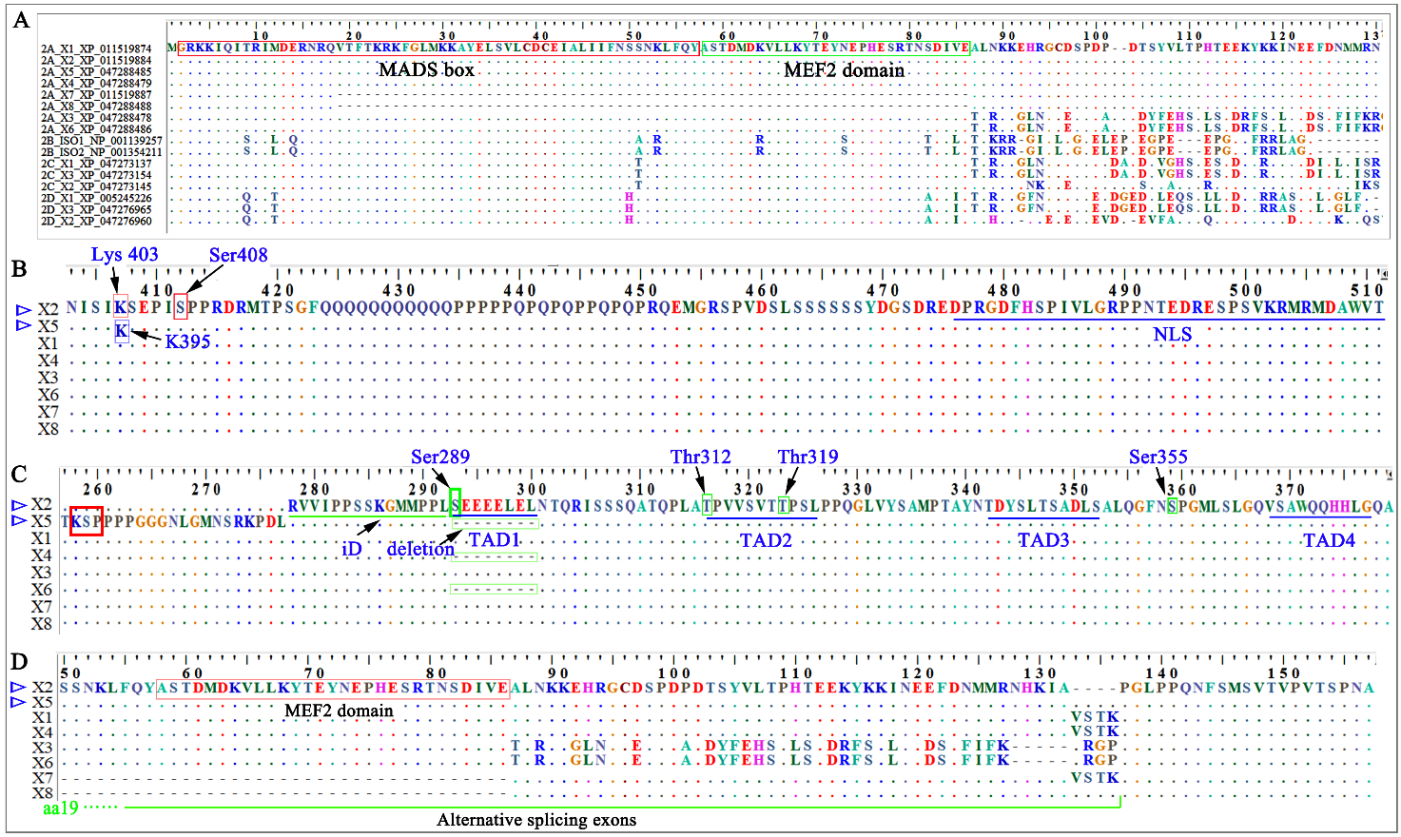
Core domains of MEF2 protein. A dot indicates that an amino acid is identical with that of the top reference sequence at the same position, and ‘-‘ indicates that a residual is deleted at the same position compared to the top reference sequence. ‘X’ plus a number represents the MEF2 variants in the NCBI database. MEF2A X2 variant (XP_011519884) is used as the reference sequence for indicating the amino acid position. (A) sequence alignment for different variants of MEF2A-D downloaded from NCBI database, ‘XP_’ or ‘NP_’ plus numbers indicate the sequence accession number in NCBI database; (B) sequence alignment for the C-termini of eight MEF2A variants; (C) KSP, transcription inhibition, transcription activation domain, and phosphorylation sites in MEF2A variants; (D) the region in which alternative splicing commonly occurs.

Myocyte enhancer factor 2 is an important family of transcription factors comprised of four members, MEF2A-D. These have an almost identical N-terminus (amino acids (aa) 1-86), but distinct C-termini (Figure 1A). The N-terminus has a MADS (MCM1, Agamous, Deficiens and Serum response factor) domain (aa 2-57) followed by a MEF2-specific domain (aa 58-86). The MADS and MEF2 domains mediate dimerization, high affinity binding to the DNA sequence CTA(A/T)4TAG/A and interaction with co-factors. The C-terminus functions mainly in transcription activation and in nuclear localization of the MEF2 proteins [1, 2]. Distinct amino acid sequences in the C-terminus of different MEF2 members may determine their specific activity of transcription activation. MEF2A is located on chromosome 15q26.3 and is composed of 12 exons with a total length of about 115 kb [3]. A region in its C-terminus and encompassing aa 472-507 harbors the key nuclear localization signal (Fig. 1B), while the aa 321-472 region is thought to be the transcriptional activation domain (TAD) [2]. TAD has four sub-domains: TAD1 (aa 289-296), TAD2 (aa 312-322), TAD3 (aa 338-348) and TAD4 (aa 364-373) (Fig. 1C). These are conserved among MEF2A, MEF2C and MEF2D and are important for the transcription activation activity [2]. The region between aa 274 and 288 is associated with repression of transcription activation and is thus an inhibition domain (iD) (Fig. 1C) [2]. Variants generated by alternative splicing are frequently observed in the MEF2 family [4]. The alignment of eight MEF2A variants downloaded from NCBI shows that alternative splicing exons are commonly involved with the MADS box, the MEF2 domain and with downstream region (aa 87-136). aa 19-86 is missing in variants X7 and X8 compared with other variants, this may be the consequence of alternative splicing (Fig. 1D). Another common alternative splicing exon encodes TAD1 and was reported as β-exon in a previous study by Zhu et al [4].

**Figure 2.**
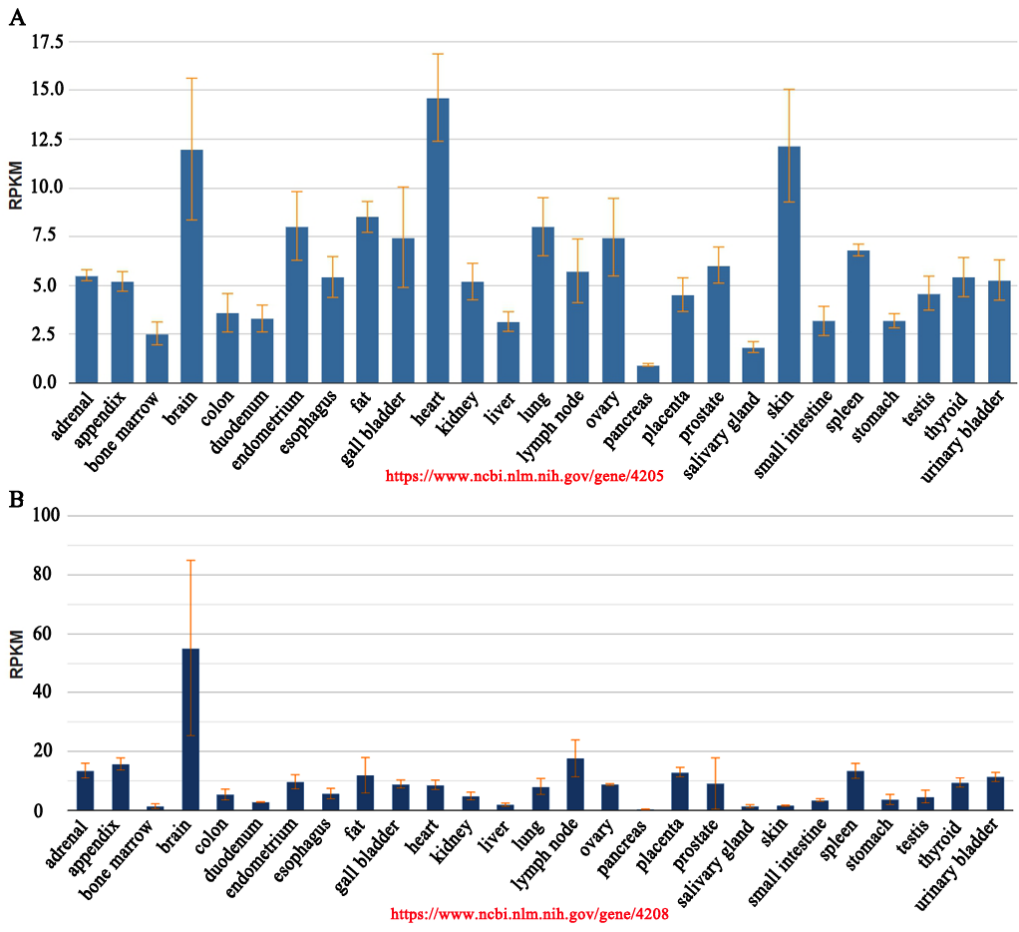
The expression of MEF2A and MEF2C in different human normal tissues determined by RNA-sequencing (Fagerberg, L. et al. 2014) [6]. RPKM, Reads Per Kilobase per Million mapped reads.

Expression of the different MEF2 members overlaps in distinct pattern during embryogenesis and in adult tissues [5]. Second-generation sequencing results of the transcriptome of various human tissues show that the highest expression of MEF2A mRNA occurs in heart tissue followed by skin and brain tissue (Fig. 2A)[6]. The highest expression of MEF2C mRNA was seen in the brain, where it was almost three-fold that of lymph nodes (second highest expression) and 6-fold that of heart (Fig. 2B)[6]. The high expression of MEF2C in brain tissue concurs with its important role in brain development. Conditional knockout of Mef2c in neuronal populations within the mouse brain causes many severe behavioral and synaptic phenotypes, while Mef2c haploinsufficiency caused by microdeletions in chromosome 5q14.3 have been linked to neurodevelopmental disorders [7]. Although redundant roles among MEF2A, MEF2C and MEF2D were observed during skeletal muscle regeneration [8], the distinct expression profiles of MEF2 members suggests that they have unique roles in different tissues in addition to their common functions. Estrella et al [9] found that MEF2A was essential for the correct differentiation of myoblasts, whereas MEF2B, MEF2C and MEF2D were dispensable for this process. Different MEF2 members can specifically recognize distinct targets amongst the total cohort of MEF2-regulated genes in the muscle genome [9]. Desjardins et al [10] found that MEF2A and MEF2D, not MEF2C, was indispensable for cardiomyocyte survival, and the major role of MEF2C was involved in cardiac morphogenesis and direct reprogramming, more than that, MEF2A and MEF2D antagonized the regulation of cell-cycle and differentiation gene programs by MEF2C. Mef2a loss of function mutations, or Mef2a knockout mice in a background of normal function for other MEF2 members, still results in an obvious disease phenotype [11, 12], indicating that MEF2A has irreplaceable functions. Indeed, dysfunction of MEF2A has been associated with cardiovascular disease, neurodegenerative disease and tumor progression [11, 13-16].

MEF2A is expressed in non-muscle cells as well as in muscle cells [17]. It is also highly expressed in myocardium, skeletal muscle, smooth muscle, neuronal cells and coronary artery endothelial cells [2, 11]. The expression pattern of MEF2A in endothelial cell precursors is similar to that of vascular endothelial growth factor receptor 2 and von Willebrand factor (endothelial cell marker) [18]. Mef2a mRNA can be detected in the blood vessels of early mouse embryos [19], while MEF2A protein can be detected in cells of the embryonic vascular system as early as 8.5 days after oocyte fertilization. These results support the notion that MEF2A plays an important role in controlling vascular morphogenesis. There are also reports that MEF2A can mediate many detrimental cellular events, including senescence, death and apoptosis [20-23]. Hence, it appears that MEF2A is widely involved in the regulation of cell proliferation, differentiation, survival, and death. However, it is still not clear how these different or opposing cell fates are regulated by MEF2A. Here, we summarize the physiological functions of MEF2A in different cells and tissue types, we also review the regulatory mechanism and regulatory network of MEF2A transcriptional activity in order to better understand the physiological functions of MEF2A.

## 2. Association of genetic variants in MEF2A with cardiovascular disease

Wang et al. [11] reported that a 21-bp deletion (△21bp-del) in the last exon of MEF2A co-segregated with coronary artery disease (CAD)/myocardial infarction (MI) in patients with a family pedigree. This study sparked the interest of many researchers in cardiovascular disease. Since then, a large number of studies have reported on the correlation between genetic variants in the MEF2A coding region and CAD/MI. In subsequent MEF2A re-sequencing studies in the Chinese Han population, the MEF2A △21bp-del was found in a small number of CAD/MI patients. However, its low population frequency means that it cannot account for the etiology of the sporadic CAD/MI [24-26]. However, several new mutations were found during MEF2A re-sequencing studies, including N263S and P279L in exon 7 [26] and a repeat number variation of CAG and CCG in exon 11 [24]. Li et al. [27] found several mutations in exon 11 of MEF2A that were unique to CAD patients, but their population frequency was low and they showed no statistical correlation with CAD susceptibility. Dai et al. [28] reported that the TT genotype of the 1353 G/T locus of MEF2A, and the haplotype of 1291-1293 CCG D + 1305 G + 1353T, may be associated with early-onset CAD. Xu et al. [29] also reported that a novel 6-bp deletion co-segregated with CAD in a family pedigree study, but this variant was not found in sporadic cases.

Weng et al. [30] re-sequenced the MEF2A gene in sporadic cases of CAD. Only one older patient was found to carry the △21bp-del mutation amongst 300 patients. Further study on 1500 individuals without CAD revealed two △21bp-del carriers. Genotyping of 19 family members of the above three probands found the △21bp-del did not co-segregate with CAD, demonstrating that it was not a genetic risk factor for CAD, at least not in Caucasians, and thus not a common cause of CAD. However, the conclusion of this study was questioned because the non-CAD individuals were determined without the use of angiography [31]. Nevertheless, studies on Japanese [32], Italian [33], German [34], Iranian [35] and Irish [36] populations also found no significant correlation between the △21bp-del in MEF2A and CAD/MI. It should be borne in mind however that the △21bp-del is rare in human populations and therefore it is difficult to reach statistical significance with limited sample size.

Reported correlations between other polymorphic loci in the exonic region of MEF2A and CAD/MI are also controversial. In the Saudi population, several SNPs located in exon 11 showed weak correlations with CAD or MI. Among these, haplotype 1A-2G-3G-4A-5C-6G-7G-8A comprised of eight SNPs was associated with a significantly increased risk of CAD (OR = 6.39) [37]. In a Spanish population, Gonzalez P et al.[38] found the 279 Leu allele frequency was three-fold higher in CAD/MI patients compared to the control group. Another Spanish population study found the CAG repeat (poly Q) polymorphism (CAG)_11_ allele of MEF2A was a risk factor for left ventricular hypertrophic cardiomyopathy, and was second only to the risk from hypertension [39]. A study of an Iranian population investigated two SNPs: rs34851361 (A/G) and rs325400 (T/G). The authors found that rs325400 (T/G) and the haplotype A-T composed of these two loci were significantly correlated with CAD susceptibility [40]. A case-control study of a Chinese population found that a CAG repeat polymorphism in exon 11 of MEF2A was associated with CAD susceptibility. The (CAG)_9_ allele frequency in CAD patients was significantly higher than in the control group (*P* < 0.001), and was also associated with the severity of CAD [41]. However, a large case-control study of a German population did not find any mutations or polymorphisms that were associated with MI, including Pro279Leu in exon 7 and the (CAG)n repeat in exon 11 [36]. In addition to the observation that the (CAG)_11_ allele was associated with the risk of left ventricular hypertrophic cardiomyopathy in the Spanish population, a heterozygous nonsense mutation (Gly240*) in MEF2A also co-segregated with dilated cardiomyopathy in a Chinese family pedigree [13]. A nonsense mutation in MEF2C (p.Y157*) was associated with dilated cardiomyopathy in another Chinese family pedigree [42]. The cardiomyopathy phenotype induced by loss of function mutations in MEF2A and MEF2C indirectly supports the important role of these genes in the development of cardiac tissue, as demonstrated also in animal models[12, 43, 44].

To date, no MEF2A genetic variation has been consistently associated with sporadic CAD/MI. Several genetic variants found in pedigree studies, which result in severe dysfunction of MEF2A have not been linked to CAD/MI susceptibility in sporadic cases. However, the frequency of these variants in the population is very low and hence it may be difficult to achieve statistical significance with a limited sample size. This also shows that mutations causing serious dysfunction of MEF2A are difficult to spread in the population, although they do occasionally occur as heterozygotes in adult individuals of some family pedigrees. In such individuals, the function of MEF2A may be compensated by other genes. These observations also indirectly show that MEF2A function is very important for the entire life process of human beings. In light of the apparent functional redundancy of MEF2, an interesting question is whether other MEF2 members have high expression levels in individuals who carry loss-of-function mutations in MEF2A and survive to adulthood.

## 3. Physiopathological functions of MEF2A

### 3.1MEF2A functions in myocardial formation, differentiation and pathology

MEF2A is necessary for cardiac development and myocardial differentiation. It is the main MEF2 gene product expressed in postnatal myocardium. Most mice that lack MEF2A die suddenly in the first week after birth and show significant right ventricular dilation, myofibril rupture, mitochondrial disorder, and fetal heart gene program activation. The few MEF2A knockout mice that survive into adulthood also show myocardial mitochondrial deficiency and susceptibility to sudden death [12]. These findings reveal specific roles for MEF2A in maintaining the appropriate mitochondrial content and cellular structural integrity of the postnatal heart, roles which cannot be compensated by other MEF2 sub-types [12]. The phenotype of Mef2a-mutant mice is clearly distinct from that of Mef2c mutants, which die from severe cardiovascular abnormalities at embryonic day 9.5 [12, 45]. This observation suggests that MEF2A and MEF2C possess distinct functions beyond their redundancy. The promoter regions of *COX6A_H_* and *COX7A_H_* lack NRF sites but have a conserved MEF2 domain. Chromatin immunoprecipitation (ChIP) assay shows that NRF1 binds to the promoter of *MEF2A*, and that MEF2A binds to the promoter of *COX6A_H_*, *PPARGC1A* and to that of *MEF2A* itself. *MEF2A* mRNA and promoter-reporter-signal are regulated by the expression level of NRF1 and show a positive association. PGC1α stimulates increased MEF2A promoter-reporter activity. This effect is mediated by the NRF1 and MEF2 elements of the MEF2A promoter, since mutation of either site results in attenuation of the PGC1α effect [46]. These results indicate that MEF2A coordinates the expression of cardiac and muscle respiratory chain subunits through the NRF1→ MEF2A→COX_H_ transcription cascade [46]. Destruction of this cascade regulatory loop may explain the lack of mitochondria in the striated muscle of MEF2A deficient mice [46]. The DNA-binding transcription factors MEF2A, NKX2.5, GATA4 and SRF play key roles in the differentiation, maturation, and homeostasis of cardiomyocytes [34]. MEF2A can binds to the Gata4 and Nkx2.5 promoters and depletion of MEF2 decreases the expression of these genes [47, 48]. Schlesinger et al [47] found that among hundreds of target genes with transcription factor binding sites (TFBS) for GATA4, MEF2A, NKX2.5 and SRF in mouse HL-1 cardiomyocytes, many overlap with and are co-regulated by at least two of the GATA4, MEF2A, NKX2.5 and SRF transcription factors. The Gene Ontology terms of 'muscle cell differentiation' and 'heart looping', are significantly over-represented amongst MEF2A and NKX2.5 targets, respectively. This indicates that MEF2A plays an independent and central role in cardiac development and function. It also functions in coordination with GATA4, NKX2.5 and SRF, and all of these factors are mutually regulated. In addition, MEF2A plays a role in cardiomyocyte survival by regulating costomere integrity [49]. Activation and elevated expression of MEF2A and MEF2C in the adult heart is often closely related to myocardial hypertrophy [50]. MEF2A increases the expression of COX-2 by up-regulating the expression of heparin-binding EGF-like growth factor (HB-EGF), which is related to cardiomyocyte hypertrophy induced by MEK5-ERK5 signal [51]. Abnormally elevated calcium signal induces myocardial hypertrophy. Calcium/calmodulin-dependent protein kinase (CaMK) is an effective activator of MEF2 activity (reviewed in ref. [5]). CaMKs promotes the expression of several fetal heart genes by increasing the transcriptional activity of MEF2A, thus inducing myocardial hypertrophy (reviewed in ref. [5]). Ventricular hypertrophy in rats exposed to arsenic trioxide may be related to the up-regulation of MEF2A, CAMKK2, CALM3 and TNNI3 in cardiomyocytes induced by this agent [52]. The long noncoding RNA Ahit prevents myocardial hypertrophy by down-regulating MEF2A expression in synergy with SUZ12 [53]. Suppressors of MEF2 (class II HDACs and MITR) are highly expressed in the heart, brain and skeletal muscle, thus mirroring the tissues in which high levels of MEF2 expression are observed (reviewed in ref. [5]). Together, the above findings suggest that maintaining a physiological level of MEF2A activity is critical, and that either hypo- or hyper-activity may lead to deleterious consequences.

### 3.2 The role of MEF2A in vascular endothelial cell function and angiogenesis

The main MEF2 members expressed in vascular cells are MEF2A and MEF2C. MEF2 protein has been shown to control vascular integrity by promoting endothelial cell (EC) survival. This is essential for EC homeostasis and for the regulation of protective genes against atherosclerosis [54]. However, Materna et al [43] found that almost complete early deletion of Mef2c in the vascular endothelium resulted in viable offspring that were indistinguishable from wild type mice and had no overt defects in their vascular system, suggesting a less important role for Mef2c in vascular development. MEF2A may therefore be more important than MEF2C for the development and normal functioning of the vascular endothelium. MEF2A is a transcriptional effector for vascular endothelial growth factor signal, and thus a key regulator of angiogenesis [55]. The expression of KLF2 in ECs is induced by many factors, including laminar shear stress and angiopoietin-1 (Ang1). The flow-dependent induction of KLF2 is mainly mediated through the MEK/ERK/MEF2 pathway [56]. However, the induction of KLF2 by Ang1 is via the PI3K/Akt/MEF2 pathway and is not mediated by the signaling pathways involving ERK5 and HDAC5 [57]. We previously found that inhibiting the expression of MEF2A in vascular endothelial cells (VECs) resulted in accelerated cell aging, and therefore MEF2A over-expression could rescue VECs from senescence induced by oxidative stress [58]. Resveratrol has been shown to delay the aging and apoptosis of VECs. Our previous study found that treatment of VECs with resveratrol significantly up-regulated the expression of MEF2A, thereby preventing apoptosis induced by oxidative stress [59]. When siRNA was used to specifically silence MEF2A and the VEC were then treated with resveratrol, the apoptosis induced by silencing MEF2A could not be reversed [59]. Knockout of MEF2A or MEF2C in human pulmonary artery endothelial cells (PAECs) results in cellular dysfunction. The transcriptional activity of MEF2 in PAECs from pulmonary artery hypertension (PAH) patients was impaired by the increased nuclear localization of HDAC4 and HDAC5. Moreover, the restoration of MEF2A activity reversed the PAH phenotype in experimental models [60]. Evidence for the protective effect of MEF2A on blood vessels also includes the observation that inhibition of MEF2A in ApoE^-/-^ mice promotes a vascular inflammatory response and atherosclerosis [59, 61]. Together, the above studies suggest that MEF2A plays an important role in maintaining the normal physiological function of VECs. However, several studies have also reported harmful effects of MEF2A on endothelium. For example, inhibition of MEF2A in the myocardium of a type 1 diabetes mouse model reduced myocardial fibrosis by partially regulating the transformation of endothelium to mesenchyme [20]. Additionally, up-regulation of MEF2A was associated with HUVEC dysfunction and oxidative stress injury induced by glucose deprivation [62, 63]. Finally, inhibition of MEF2A enhanced neovas-cularization and promoted blood flow recovery after ischemia in vivo via post-transcriptional regulation of the miR-329 and miR-494 microRNAs [64]. The above studies demonstrate the dual and opposing roles of MEF2A in vascular endothelium and vascular tissue, while highlighting the complex regulation of vascular endothelial function by MEF2A. More in-depth research is needed to fully understand the mechanisms by which MEF2A mediates different vascular endothelial functions.

### 3.3 Role of MEF2A in the proliferation, differentiation, and pathology of smooth muscle and skeletal muscle

Regeneration of skeletal muscle requires the participation of MEF2A, MEF2C and MEF2D [8]. A study reported that MEF2A can antagonize the expression of Hspb7 in mouse skeletal muscle and this may benefit preventing muscle atrophy [65]. MEF2A promotes the proliferation and differentiation of myoblasts through direct transcriptional regulation of myozenin2 (MYOZ2) [66]. It also participates in myocyte differentiation through direct regulation of calpain 3 (Capn3) [67], and plays a role in myofibrogenesis by regulating the expression of Myospryn [68]. Depending on the p38 and calmodulin activity levels, static stretch promotes the nuclear localization of MEF2A in C2C12 myocytes, thereby inducing the expression of myosin heavy chain (MHC) [69]. However, MEF2A appears to have different physiological functions in vascular smooth muscle cells (VSMC). Treatment of VSMCs with H_2_O_2_ promotes cell senescence by inducing significant up-regulation of MEF2A [21]. However, the opposite result is obtained after H_2_O_2_ treatment of VECs, wherein it promotes cellular senescence by significantly down-regulating MEF2A [58, 70]. Inhibition of MEF2A induces the phenotypic transformation, proliferation, and migration of VSMC [71]. Reported pathological factors that induce MEF2A up-regulation in VSMC also include AngII and balloon injury [72, 73]. The different functional roles observed for MEF2A in VECs and VSMCs has resulted in considerable confusion regarding its importance in vascular diseases, especially atherosclerotic vascular diseases.

### 3.4 The role of MEF2A in neuron survival, differentiation, and neuro-degenerative diseases

Accumulating evidence points to an important physiological function for MEF2A in the nervous system. MEF2A and MEF2D are highly expressed in hippocampal CA1 neurons, while MEF2C expression is located mostly in the dentate gyrus and neocortex [74]. Redundant function between MEF2A and MEF2D was revealed in granule neurons, as one of them loss function, that will be at least partially compensated by the other [75]. MEF2A plays a key role in the differentiation and maturation of rat neural stem cells into neurons, while also being necessary for the survival of cerebellar granule neurons. Chromatin immunoprecipitation (ChIP) sequencing has revealed that MEF2A-specific binding sites in mouse neuronal cells were mainly enriched in MAPK signaling pathway, axon guidance and oxytocin signaling pathway [76]. Specific silencing of MEF2A significantly reduces MEF2 response element-mediated transcription in granule neurons and inhibits the survival of granule neurons [77]. Phosphorylation-dependent degradation of endogenous MEF2A in rat cerebellar granule neurons induces neuronal apoptosis [78, 79]. Neurotoxin-induced granule neurons apoptosis is caused by caspase-dependent degradation of MEF2A and MEF2D, not of MEF2C, that are phosphorylated by cyclin-dependent kinase 5 (CDK5) [80]. Under physiological conditions, MEF2A is degraded through chaperone-mediated autophagy (CMA). This mechanism can protect primary neurons from oxidative stress-induced cell damage. Under pathological conditions, mild oxidative stress (200 μM H_2_O_2_) increases the degradation and activity of MEF2A. Excessive oxidative stress (> 400 μM H_2_O_2_) halts its degradation, resulting in the accumulation of non-functional MEF2A and harmful effects on cells [81]. By inhibiting the activity of proteasome, neurotoxin stops the degradation of ubiquitin-modified MEF2A and the accumulation of inactive MEF2A in the nucleus, thereby inducing cell damage [82]. These indicate that the decreased expression, the disruption of the functional MEF2 and the accumulation of function-impaired MEF2 are pernicious for neuron survival. The functional role of MEF2A, MEF2C and MEF2D in the cellular and non-cellular autonomous control of adult hippocampal neurogenesis is different to their role in the process of neural development. Although the number of induced neurons in MEF2 knockout mice was increased, the dendrites poorly developed, demonstrating a lack of coupling between neuronal survival and dendritogenesis [83].

The expression of MEF2A and the regulation of its activity both play a role in neuronal differentiation. MEF2A is highly expressed in differentiated neurons in the brain throughout synaptogenesis [74, 84, 85], and plays a key role in neural presynaptic development in mammals. Down regulation of MEF2A in vivo significantly increases the density of orphan presynaptic sites in primary neurons and rat cerebellar cortex [86]. However, covalent modification of MEF2A at lysine 403 by small ubiquitin related modifier (SUMO) protein inhibits the activity of MEF2A and induces the postsynaptic dendritic differentiation in the mammalian brain [84]. The sumoylated transcription inhibitor form of MEF2A mediates the inhibition of orphan presynaptic sites. Synaptic aggregation protein 1 (Syt1) is a direct inhibitory target gene of sumoylated MEF2A in neurons. Inhibition of Syt1 eliminates the role of orphan sites downstream of MEF2A. The elimination of orphan presynaptic sites induced by MEF2A triggers the maturation of presynaptic boutons in the brain [86]. MEF2A has been implicated in the morphogenesis of dendritic claws of granule neurons in the cerebellar cortex [84, 85]. The modification of MEF2A required for postsynaptic differentiation occurs via a phosphorylation-regulated sumoylation to acetylation switch (SAS) peptide motif, which activates MEF2A and in turn inhibits dendritic claw differentiation [87, 88]. MEF2C, but not MEF2A and -D, plays major role in hippocampal synaptic function [89]. However, observation that sudden discharge induces the expression of MEF2A/D-dependent target gene Arc that contributes to synaptic silencing and elimination indicates involvement of MEF2A/D in synaptic function [90].

The plasticity thresholds of brain neurons during neural circuit formation are fine-tuned via altering MEF2 levels [91]. Abnormal expression and functional defects of MEF2A are often associated with neurodegenerative diseases. Hypermethylation of the MEF2A promoter [14] or genetic variants in MEF2A exons [15] have been linked to Alzheimer's disease. Experiments with a mouse model show that deletion of *Mef2a* can lead to behaviors related to autism and drug addiction [92, 93]. The neuropeptide oxytocin (OT) induces neurite contraction in neurons that express normal levels of MEF2A and neurite growth in neurons that lack MEF2A. Therefore, MEF2A is believed to determine the responsiveness of neuronal cells to OT and to be involved in the pathological mechanism of autism [94, 95]. MEF2A may also regulate the expression of genes related to thought disorders. Mutations in the MEF2 binding sites of the two schizophrenia related genes QKI (HGNC: 21100) and PDE10A (HGNC: 8772) have been associated with formal thought disorder [96]. Halting the degradation of the ubiquitinated MEF2A by neurotoxins induces the characteristics of Parkinson's disease in an animal model [82], indicating that accumulation of inactive MEF2A is also one of the causes of neurodegenerative diseases. Degenerated cognitive ability is one of the hallmarks of neurodegenerative diseases. Clinical brain transcriptomic data shows the expression level of MEF2C is positively correlated with cognitive ability, while over-expression of MEF2A/C in PS9 mice remarkably improves the cognition and reduces hyperexcitability [97]. MEF2A also regulates the bidirectional interference of activity-dependent AMPA receptors, which constitute a major foundation of learning and memory [98]. However, two studies reported that MEF2A is associated with reduction of the ability for spatial learning and memory in mice [93, 99]. These findings about the roles of MEF2 in cognition and learning abilities seem to be contradictory. Since the expression of MEF2A promotes neurite contraction and reduces hyperexcitability, is the negative correlation between MEF2A and spatial or sport learning abilities observed in mice caused by the increase of neural hyperexcitability resulted from reduction of MEF2 expression?

The protective effect of some drugs on cerebral ischemia injury was shown to depend on an increase in MEF2A activity. Dexmedetomidine combined with netrin-1 can reduce cerebral ischemic injury by inhibiting endoplasmic reticulum stress via activation of the ERK5/MEF2A pathway [100]. Moreover, alternative splicing of MEF2A is associated with some neurological diseases. Significant differences have been reported in the MEF2A mRNA splices observed in muscle tissues from patients with myotonic dystrophy (DM) and neuromuscular disorder (NMD) compared to normal muscle tissue [101].

The current evidence indicates that a normal level of MEF2A expression and activity is necessary for the development, differentiation and survival of neurons, as well as for communication in the nervous system. MEF2A is an important regulator that ensures the health of the nervous system. All of these highlight the potential of MEF2A as therapeutic target or biomarker for neurodegenerative disease.

### 3.5 The role of MEF2A in tumors

MEF2 can support oncogenic or tumor suppressive activity, depending on its interactions with co-activators or with co-suppressor chaperones. In leiomyosarcoma (LMS), the expression level of class II HDACs determines whether MEF2 plays a tumor inhibitory or promoting role. Knockout of HDAC9 inhibits the transformed phenotype of LMS cells by restoring the transcription of some MEF2 target genes [102]. Transforming growth factor (TGF)-β plays a role in the invasion, metastasis and growth of cancer cells by promoting HDAC degradation and histone hyper-acetylation at the MEF2 site of the matrix metalloproteinase (MMP-10) promoter, and by activating MEF2A and up-regulating the expression of MMP-10 [103]. MEF2A expression is thought to promote the progression of multiple myeloma [104] and of colorectal cancer [105], with higher expression correlating with poor prognosis. P38 MAPK activation in the gastric cancer cell line MKN45 up-regulates GLUT-4 in a MEF2A-dependent manner and promotes glucose uptake and cell growth [106]. This suggests that a high expression level for MEF2A promotes the growth and metastasis of gastric cancer cells. However, other studies have reported tumor suppressive activity for MEF2A. For example, epigalocatechin-3-gallate (EGCG) promotes the expression of KLF4 via MEF2A, thereby inhibiting the growth of gastric cancer cells [107]. Chen et al [108] found that MEF2A transcriptionally activates expression of the lncRNA HCP5, thus inhibiting the progression of gastric cancer. Houttuynia cordata was reported to induce the apoptosis of human HepG2 hepatoma cells by activating the HIF1a-FOXO3 and MEF2A pathways [109]. Clearly, additional research is required to allow more accurate conclusions about the role of MEF2A in tumor development and progression. The studies to date have investigated only one aspect of the activation and regulation pathway of MEF2A. However, MEF2A is part of a complex regulatory network, and its activity for transcriptional activation is affected by many factors including its expression level, protein modification and interacting co-factors. The final physiological and pathological roles of MEF2A are likely to be the result of multi-dimensional synergies or competitions.

### 3.6 Other physiological roles

The observation that Mef2a-knockout female instead of male mice are osteopenic suggests the role of MEF2A in osteoclast differentiation is associated with sex [110]. Suppression of MEF2 signal was reported to inhibit activation of human primary hepatic stellate cells [111]. MEF2A is expressed much higher than other three MEF2 members in primary human cytotrophoblasts, and induces cytotrophoblast differentiation and syncytium formation, suggesting dysregulation of MEF2A may be associated with placenta-related pregnancy disorders [112]. Negative role of MEF2A against survival of retinal ganglion cells was observed in mice after optic nerve crush [22]. These suggest that MEF2A may have many unique but distinct from the well-studied physiological functions in different tissues.

## 4.MEF2A: regulation of activity and regulatory targets

### 4.1 Regulation of MEF2A activity by post-translational modifications

Post-translational modifications are an important way to regulate MEF2A activity, with phosphorylation, acetylation and sumoylation being the most common. MEF2 protein is thought to be a nuclear target of the signal cascade in response to serum stimulation and to some cellular stressors [113]. Its transcriptional activity is increased following phosphorylation by p38 MAPK, and to a lesser extent after phosphorylation by the PKC isoforms δ and ε [113]. Depending on the sites, phosphorylation of MEF2A can either inhibit or enhance its activity. Ser255 in MEF2A (variant X2, Figure 1C) is located within an evolutionarily conserved KSP motif and contains phosphorylation receptor sites for MAPK members (ERK1, ERK2 and stress activated protein kinase), Cdc2-like kinase (including Cdk5) and glycogen synthase kinase-3 (GSK3). The phosphorylation of Ser255 in MEF2A is independent of p38 MAPK, while the KSP motif is important for regulating the stability and function of MEF2A. The neuronal apoptosis induced by GSK3 may be related to excessive degradation of MEF2A following phosphorylation of Ser255. Thr312 and Thr319 in MEF2A are p38 MAPK-dependent phosphorylation sites (Fig. 1C). Their phosphorylation markedly increases the transcriptional activity of MEF2A [114]. ERK5 phosphorylation of MEF2A also increases its transcriptional activity [115]. GSK3β can inhibit p38 MAPK activity. Drugs that suppress GSK3β therefore attenuate the inhibition of p38 MAPK activity and promote the transcriptional activity of MEF2A/D in skeletal muscle and cardiomyocytes [116], thus demonstrating MEF2 activation through phosphorylation by p38 MAPK. The epidermal growth factor (EGF)-induced activation of MEF2A is specifically mediated by big mitogen-activated protein kinase (BMK1) that increases the transcriptional activation activity of MEF2A via phosphorylating its Ser-355, Thr-312 and Thr-319 [117]. Ser289 in MEF2A is a protein kinase CK2 site (Figure 1C). There is also a homologous region of this CK2 site in MEF2C and it is a known alternative splice site in different tissues [114]. The region surrounding this site could therefore determine the specific functional role of MEF2 in different tissues. Phosphorylation and dephosphorylation of Ser408 in MEF2A (Fig. 1B) is one of the switches that control the transition between inhibition and activation of MEF2A [22]. In the lower organism Xenopus laevis, anterior formation during development depends on the phosphorylation-based activation of MEF2A transcriptional activity by Nemo-like kinase (NLK) [118].

Some studies have shown that acetylation of MEF2 is associated with its DNA binding and transcriptional activity [119, 120]. A high acetylation level of MEF2A enhances its DNA binding activity in skeletal muscle. Exercise increases the acetylation level of MEF2A, thereby increasing its’ binding to the muscle carnitine palmitoyl transferase 1b (*Cpt1b*) promoter and promoting its transcription [121]. Exercise increases the acetylation level of MEF2A largely by promoting the phosphorylation of HDAC5. This results in its translocation from the nucleus to the cytoplasm, hence reducing its interaction with MEF2A [121]. Calmodulin promotes the acetylation of MEF2A at Lys403 (Fig. 1B) through the dephosphorylation of Ser408, thereby enhancing MEF2A transcriptional activity [87].

Sumoylation of MEF2A leads to inhibition of its activity. Decreased transcriptional activity caused by sumoylation of K395 in MEF2A (variant X5, Fig. 1B) has been demonstrated both *in vivo* and *in vitro*. The nuclear E3 ligase PIAS1 was reported to promote sumoylation of MEF2A [122], while SUMO-specific proteases (SENPs) are thought to function by removing sumoylation of MEF2A. SENP2, a member of the SENPs, removes MEF2A sumoylation, thereby attenuating the inhibition of MEF2A activity [70]. Desumoylation of MEF2A may be very important in maintaining the functional role of MEF2A during embryonic development [87].

Different reversible modifications can regulate the activity of MEF2A for transcriptional activation. This allows MEF2A to have different functional roles in different tissues or cells, as well as showing large differences in the response to different stimuli in the same cell.

### 4.2 Effect of co-factors on MEF2A activity

MEF2A binds to specific MEF2 elements and interacts with many co-factors in order to activate gene transcription, thereby showing both promoter specificity and cell specificity [123]. The most typical MEF2 co-factors are muscle-specific transcription factors and chromatin remodeling enzymes, including histone acetyltransferases (HATs) and histone deacetylases (HDACs). Developmental and stress signals can positively or negatively regulate MEF2 activity by controlling its phosphorylation state and its association with co-factors (reviewed in ref. [5]). Class IIa HDACs are the most well studied inhibitors of the transcriptional activity of MEF2A. The interaction between HDACs and MEF2A in the nucleus reduces the DNA binding activity of MEF2A. HDAC5 can interact with MEF2A both *in vivo* and *in vitro* to strongly inhibit its ability for transcriptional activation [124]. The inhibition of MEF2A activity by HDACs can be attenuated by promoting their degradation and translocation from the nucleus to the cytoplasm. Apelin-APJ signaling mediates phos-phorylation of HDAC4 and HDAC5 to promote their nuclear export, thus activates endothelial MEF2A and MEF2C [125]. Calcium/calmodulin dependent protein kinase (CaMK) phosphorylates two conserved serine residues at the N-terminal of HDAC-4, -5, -7 and -9 (reviewed in ref. [5]). Phosphorylation of these residues creates a docking site for the intracellular chaperone 14-3-3, which destroys the HDAC-MEF2 complex by binding to phosphorylated HDACs and releasing MEF2 from inhibition (reviewed in ref. [5]). HDACs suppress the transcription of gene promoters by deacetylating the N-terminal tails of core histones to promote chromatin condensation. This inhibition can be attenuated by HATs (such as p300 and PCAF) that make the chromatin relax through acetylation of the N-terminal tails of core histones (reviewed in ref. [5]). The MEF2A/D:P300 complex increases the expression of C-Jun in macrophages, but the activity of MEF2A/D is suppressed when it is bound to HDAC1 and HDAC7. MEF2A/D can therefore have a dual role in the differentiation of monocytes into macrophages through its binding to different co-factors [126].

Mammalian spinous homologous protein 1 (MASH1) is necessary for the early development of the nervous system. MASH1 can regulate the expression of specific genes essential for neuronal differentiation through its synergistic effect with MEF2A. MEF2A and MASH1 are simultaneously induced during neuronal lineage differentiation. In transient transfection experiments, MEF2A and MASH1 act synergistically to activate gene expression through a mechanism based on specific physical interaction between these two factors [127]. Chicken ovalbumin upstream promoter-transcription factor II (NR2F2, COUP-TFII) is found in the mouse Leydig cells, and can form a complex with MEF2A, which binds to Akr1c14 gene promoter to potentiate its expression [128]. Early growth response-1 (EGR1) is also a MEF2 co-regulator and interacts with MEF2A to specifically repress its activity. This may be the mechanism to maintain costamere gene expression at levels commensurate with cardiomyocyte contractile activity [129].

### 4.3 Regulation of MEF2A expression

The activity of MEF2A for transcriptional activation is regulated by post-translational modifications and co-factors, as well as by transcriptional and translational regulation of MEF2A. Mitochondrial specific nuclease (*EndoG*) is thought to be a negative regulator of *Mef2a* expression, while protein kinase B2 (AKT2) promotes *Mef2a* expression by inhibiting EndoG [130]. Bone morphogenetic protein-2 (BMP-2) promotes the expression of sarcomeric myosin heavy chain (MHC) and Mef2a by increasing PI3K activity. Moreover, ectopic expression of Mef2a can increase the transcription of BMP-2 depending on the level of PI3K activity, suggesting the existence of a regulatory loop that involves BMP-2/PI3K/MEF2A/PI3K/BMP-2 [131]. The expression of MEF2A may also be self-regulating, since its promoter region contains MEF2 binding sites. In addition, phosphorylation-induced activation of MEF2A by ERK5 and p38 MAPK promotes the expression of MEF2A [132]. Exercise increases the expression of *Nrf*-1 and enhances the activity of CaMK II, resulting in hyper-acetylation of H3 histone. This causes increased transcription of *Mef2a* by enhancing the binding of NRF1 to the *Mef2a* promoter [133]. MEF2A has a very long 3'-untranslated region (3'-UTR) which contains many miRNAs binding sites that play a cis-regulatory role in translational inhibition [134]. Many miRNAs that inhibit the expression of MEF2A have been identified. MiR-155 for example inhibits the expression of MEF2A, while the lncRNA malat1 up-regulates the expression of MEF2A by adsorbing miR-155, thus promoting cardiomyocyte proliferation [135]. miR-144-3P was shown to have anti-tumor properties by inhibiting the expression of MEF2A [104], while miR-194-5p delays the progression of sepsis by down-regulating MEF2A [136].

The expression of MEF2A is also affected by various chemical agents. In the diabetes model of insulin deficiency induced by streptozotocin, the expression of *Mef2a* in heart and skeletal muscle decreases significantly [137]. Decreased expression of Mef2a and Mef2d in myocardium of rat is induced by chronic ethanol exposure [138], and the reduction of their expression in adipose-derived mesenchymal stem cells is induced by treating with indoxyl sulfate [139]. In contrast, resveratrol significantly up-regulates the expression of MEF2A in vascular endothelial cells [59]. Although regulation of the expression of MEF2A is the most direct way to control its activity for transcriptional activation, research in this area is still very limited and the relevant regulatory mechanisms have yet to be identified.

### 4.4 Downstream regulatory pathways and target genes of MEF2A

It was initially thought that downstream target genes that are transcriptionally regulated by MEF2A were mainly myogenesis-related genes. However, more recent studies have shown that MEF2A also regulates gene expression related to cell proliferation, development, survival and apoptosis [56]. The link between MEF2 and cell proliferation was shown by the finding that MEF2 regulates serum-induced c-Jun expression, which in turn regulates the cell cycle [140]. The MEF2 binding site in the c-Jun promoter is a common MEF2A/D motif that regulates cell proliferation in many cell types [140, 141]. The target genes regulated by MEF2A vary in different tissues and cell types, depending mainly on the local co-factors or MEF2A modification status. For example, in the atrium, MEF2A regulates genes involved in fibrosis and adhesion, whereas in the ventricle it controls genes related to inflammation and endocytosis [142]. In mouse myocardium, MEF2A regulates the expression of a large number of genes linked to costamere integrity and function [49]. Multiple MEF2 motifs occur in the core promoter regions of PIK3CA and PIK3CG. We previously reported that MEF2A up-regulates the expression of PIK3CA and PIK3CG, which may be directly regulated by the binding of MEF2A to their promoters [58].

MEF2A positively regulates the expression of several important transcription factors including KLF4 [60, 107], KLF2 [21, 57, 60], GATA4 [48] and NKX2.5 [47], thereby controlling major cellular processes such as proliferation and differentiation. Vascular endothelial protective genes, such as KLF2 and KLF4, are transcriptionally activated by MEF2A [54]. MEF2A may also be involved in muscle regeneration and differentiation via transcriptional regulation of microRNA (miRNA) expression. It promotes skeletal muscle regeneration by directly regulating the largest known mammalian miRNA cluster that encodes a subset of the Gtl2-Dio3 miRNAs. Gtl2-Dio3 miRNAs such as miR-410 and miR-433 suppress the WNT signaling inhibitors called secreted Frizzled-related proteins (sFRPs) [143]. Gtl2-Dio3 miRNAs are also referred to as maternally expressed 3 (MEG3)-iodothyronine deiodinase 3 (DIO3) miRNAs. These are up-regulated by MEF2A to promote bovine skeletal muscle differentiation by inhibiting protein phosphatase 2A (PP2A) signaling [144].

**Table 1:** The downstream target genes regulated by MEF2A.

Gene names	Tissue/cell	Physiopathological roles	References
KLF2	Vessel/endothelial cell	Vascular quiescence	54, 56,57
KLF4	Vessel/endothelial cell	Vascular protection	54,107
GLUT-4	Muscle, adipo	Glucose metabolism	121,137,145-152
Gata4	Cardiomyocytes	Maturation and homeostasis of Cardiomyocytes	47,48
c-Jun	Macrophages and others	Cell proliferation	126,139,140
Cpt1b	Skeletal muscle	β-oxidation of mitochondrial fatty acids	121
PIK3CA	Endothelial cell	Delay senescence	58
PIK3CG	Endothelial cell	Delay senescence	58
ZEB2	Colorectal cancer	Tumor formation and metastasis	105
CTNNB1	Colorectal cancer	Tumor formation and metastasis	105
Capn3 (calpain 3)	L6 myoblasts	Myoblast differentiation	67
COX6A_H_	Striated muscle	Mitochondrial defects	46
PPARGC1A	Striated muscle	Mitochondrial defects	46
HB-EGF	Cardiomyocyte	Cardiomyocyte hypertrophy	51
MYOZ2 (myozenin2)	Myoblasts	Differentiation	66
Myospryn	Striated muscle	Myofibrogenesis	68
MHC (Myosin heavy chain)	C2C12 myocytes	Differentiation	69
MMP-10	Mammary epithelial cells	Invasion, metastasis and growth of cancer cells	103
Akr1c14	Mouse Leydig cells	Male reproductive development and health	128
Nkx2.5	Cardiomyocytes	Differentiation, maturation and homeostasis	47
p75^NGFR^	Myocytes	Myogenic differentiation	153
Dusp6	C2C12 myocytes	Myocyte proliferation	154
Gtl2-Dio miRNA cluster	Rat skeletal muscle	Promote regeneration	143
MEG3-DIO3 miRNA cluster	Bovine skeletal muscle	Promote differentiation	144
lncRNA HCP5	Gastric cancer	Inhibits the progression of gastric cancer	108

Solute carrier family 2 member 4 (SCF2A4), also known as glucose transporter 4 (GLUT4), is the most studied MEF2A target gene. The transcriptional activation of GLUT4 by MEF2A is affected by multiple factors. CaMK promotes the expression of GLUT4 by promoting H3 acetylation and increasing the binding of MEF2A at the MEF2 site in the GLUT4 promoter. This pathway mediates coffee- and exercise-induced expression of GLUT4 [145-147]. Exercise-induced up-regulation of GLUT4 may also depend on the exercise-induced expression of MEF2A. Silva et al [148] found that up-regulation of GLUT4 induced by muscle contraction *in vitro* is associated with increased expression and binding activity of MEF2A/D. Dimerization of the GLUT4 enhancer factor (GEF) enhances its affinity for hypo-phosphorylated MEF2A. Binding of MEF2A to the GLUT4 promoter increases the binding of GEF to domain I, thereby increasing the transcriptional expression of GLUT4 [149]. In the absence of MEF2 protein, the transcription co-repressor HDAC5 can interact with GEF and specifically inhibit GLUT4 promoter activity [149]. AMP-activated protein kinase phosphorylates GEF to promote nuclear translocation of it and MEF2A, thus enhances them binding to GLUT4 promoter and increases the transcription of GLUT4 [150]. In primary dispersed adipocytes, hyper-phosphorylation of MEF2A can enhance its binding to MEF2D, thus reducing its binding to the GLUT4 promoter and resulting in down-regulation of GLUT4 [114, 151]. Expression of GLUT4 in striated muscle depends on the insulin-induced up-regulation of MEF2A [137]. However, over-expression of MEF2A in adipocytes does not affect the expression of GLUT4, nor is it sufficient to prevent down-regulation of GLUT4 in the state of insulin deficiency [152].

The exercise-induced increase in the activity of MEF2A for transcriptional activation promotes the expression of its target genes and plays an important role in cell metabolism. MEF2A promotes glucose transport and maintains the normal physiological functions of glucose metabolism by up-regulating the expression of GLUT4. CPT1B plays an important role in the β-oxidation of mitochondrial fatty acids in skeletal muscle. On one hand, exercise training induces the acetylation of MEF2A, thus enhancing its DNA binding ability. On the other hand, exercise training decreases the binding of HDAC3 and HDAC5 to MEF2A, thereby increasing its activity for transcriptional activation. Exercise therefore increases the binding of MEF2A to the promoter region of Cpt1b and enhances its transcription [121]. The above studies highlight the important regulatory roles played by MEF2A in various metabolic pathways.

Direct inhibition of gene promoter activity by MEF2A has also been reported. The down-regulation of p75^NGFR^ expression triggered by cell density phenomenon prior to the start of myogenic differentiation leads muscle progenitor cells to their ultimate fate. A mechanism is needed to counteract the effect of MyoD on p75^NGFR^ promoter activity. High expression levels of MEF2A abolish the MyoD-induced p75^NGFR^ promoter activity, suggesting that cell-specific regulation of the p75^NGFR^ gene may depend strictly on the intracellular composition and balance of appropriate bHLH transcription factors and their regulators [153]. This result shows that MyoD and MEF2A mediate the activation and inhibition of the p75^NGFR^ gene during muscle development. One possible mechanism is that MEF2A binding to MyoD occupies or affects the bHLH domain of MyoD, thus preventing MyoD from binding to the p75^NGFR^ promoter. The activity of dual specificity phosphatase 6 (Dusp6) promoter in C2C12 cells can be suppressed by MEF2D binding depending on p38MAPK [154]. These show the role of MEF2 as a direct suppressor.

**Figure 3.**
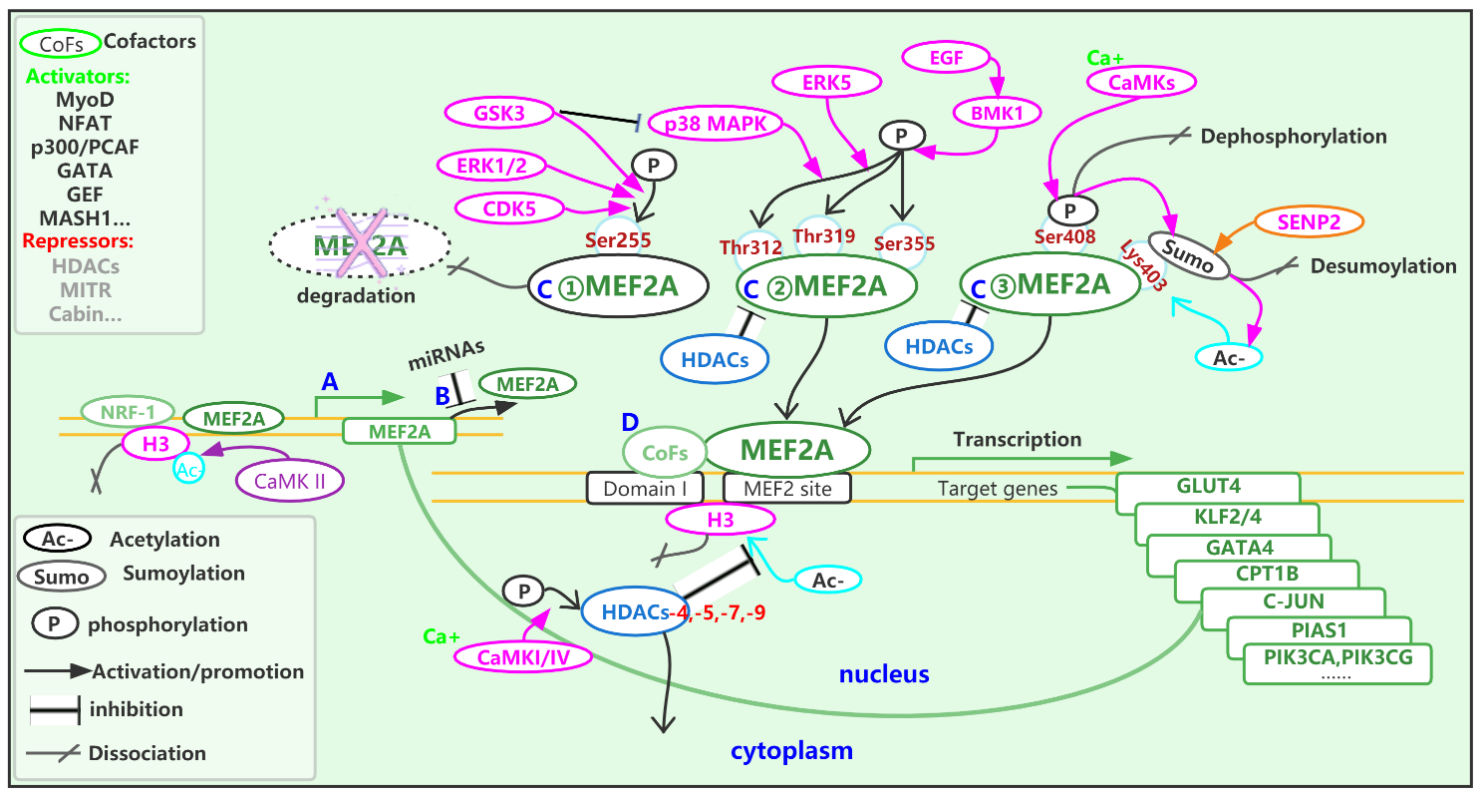
The regulatory network involved in MEF2A. The activity of MEF2A for transcriptional activation are regulated on several ways, including (A) transcription, (B) translation, (C) post-translational modification, and (D) interaction with co-factors. H3, core histone H3; Ca^+^, calcium ion; CaMK, calcium/calmodulin-dependent kinase; HDACs, histone deacetylases; Ser255 and Ser355, Serine residue in MEF2A at position 255 and 355, respectively; Thr312 and Thr319, Threonine residue in MEF2A at position 312 and 319, respectively; Lys403, Lysine residue in MEF2A at position 403 and 395, respectively.

In mammals, thousands of genes contain MEF2 motifs, making them potential targets for MEF2A. However, the increased expression of MEF2A alone does not necessarily lead to a change in the expression of its target genes. Many other factors can affect the transcriptional activation of target genes, including the modification, degradation and activation of MEF2A itself, and the association and dissociation of MEF2A with co-activators or co-inhibitors. In addition, condensation and relaxation of the chromatin surrounding the promoter regions are affected by the acetylation status of core histones (Fig. 3). The multi-layered regulation and control of MEF2A activity for transcriptional activation means that MEF2A can regulate opposing cell physiological processes. Furthermore, MEF2A can have completely opposite functional roles in different cells or tissues. Figure 3 shows the main regulatory pathways or molecules involved in MEF2A-mediated transcriptional activation, together with the target genes regulated by MEF2A. Table 1 indicates the reported MEF2A target genes and their physiological or pathylogical roles.

## 5. Conclusion

Under physiological conditions, MEF2A usually acts as a cytoprotective factor to promote the proliferation, differentiation and survival of cardiomyocytes, vascular endothelial cells, smooth muscle cells and neuronal cells. MEF2A is essential for the formation of mitochondria in cardiomyocytes. However, under certain pathological conditions, MEF2A may also have detrimental functions by promoting SMC aging, myocardial hypertrophy and fibrosis, and tumor progression. Depending on the co-factors that it interacts with, MEF2A can promote either tumor inhibition or progression. The activity of MEF2A for transcriptional activation is determined by its expression level, genetic variation, alternative splicing, post-translational modification (eg. phosphorylation, acetylation, sumoylation), and interaction with co-factors. This multi-layered regulation establishes a central role for MEF2A in regulatory networks and in multifaceted physiological functions.
